# Host Response of Atlantic Salmon (*Salmo salar*) Re-Inoculated with *Paramoeba perurans*

**DOI:** 10.3390/microorganisms9050993

**Published:** 2021-05-05

**Authors:** Michelle McCormack, Anita Talbot, Eugene Dillon, Ian O’Connor, Eugene MacCarthy

**Affiliations:** 1 Marine and Freshwater Research Centre, Galway Mayo Institute of Technology, Dublin Road, H91 TRNW Galway, Ireland; Anita.Talbot@gmit.ie (A.T.); ian.oconnor@gmit.ie (I.O.); Eugene.McCarthy@gmit.ie (E.M.); 2 Conway Institute of Biomolecular & Biomedical Research, University College Dublin, Dublin 4, Ireland; eugene.dillon@ucd.ie

**Keywords:** amoebic gill disease, immune response, gill proteome, repeat inoculation

## Abstract

In aquaculture, recurrence rates of amoebic gill disease (AGD) caused by the ectoparasite *Paramoeba perurans* are high and no prophylactic strategies exist for disease prevention. In this study, Atlantic salmon (*Salmo salar*) were initially inoculated with *P. perurans* and following the development of amoebic gill disease were treated with freshwater immersion on day 21 and day 35 post inoculation. Fish were re-inoculated following a negative qPCR analysis for the presence of *P. perurans*. The gill host immune response was investigated at 7, 14, and 18 days post re-inoculation. Differential proteome expression of immune related proteins was assessed by comparison of each time point against naïve controls. In the gill, some proteins of the innate immune system were expressed in response to gill re-colonization by *P. perurans*, while no features of adaptive immunity were found to be differentially expressed. Many of the proteins identified are novel in the context of AGD and their expression profiles suggest that their roles in the response to disease development and progression in single or multiple infections warrant further investigation.

## 1. Introduction

First reported in 1986 [1], the incidence of amoebic gill disease (AGD), an ectoparasitic condition caused by the protozoan parasite *Paramoeba perurans*, has escalated to become one of the most significant health threats in Atlantic salmon (*Salmo salar*) farming globally [2]. Typical features of AGD include multifocal gill hyperplasia along with lamellar fusion, increased mucus production and macroscopic lesions on gill filaments [3,4]. Affected fish experience respiratory distress, and mortality rates of up to 80% in untreated cases have been reported [5]. No prophylactic measures currently exist, and treatments are limited to freshwater or hydrogen peroxide baths, which remove the amoeba from affected gills and reduce gill mucus viscosity [3,6]. While the rate of re-infection has been reported to be delayed by hydrogen peroxide treatment when compared to fresh-water treatment [7,8], neither treatment prevents re-infection and repeated treatments may be required within one life cycle. Therefore the economic impact of AGD is not insignificant [9].

The high rate of AGD re-infection reported in aquaculture has prompted investigation of the host adaptive response in the case of recurrent infection. Many studies investigating gene expression after single infection have highlighted a transcriptional downregulation of key immune factors, such as interferon induced MHC I and MHC II pathways and anti-oxidant enzymes [10,11], while others reported upregulation of pro-inflammatory cytokine and immune-regulatory gene transcription [12,13,14,15] as a typical response to this infection. Those studies which investigated AGD re-infection yielded similarly disparate findings. Valdenegro-Vega et al. [16] reported no significant differences in antibody gene expression in salmon plasma or skin following four successive infections, when compared to naïve controls, and only lesion specific upregulation of IgM was detected in the gill 31 days after infection exposure. Conversely, Pennacchi et al. [17] detected a slight downregulation of IgM, IgT, and TCR mRNA in gills. Contradictory reports on the host immune response may be explained, at least for gill tissue, by differences in cellular composition and by the discrepancies commonly observed when comparing data reported from lesioned versus non-lesioned tissue [14,15,18,19]. Furthermore, the stage of disease progression has also been shown to influence immune marker expression, as evidenced by IgM elevation observed in moderately severe lesions, being absent in severe lesions [18].

In an attempt to elucidate the mechanisms underlying the host response to repeat infection with *P. perurans*, this study aimed to analyze the gill proteome of salmon experiencing re-infection in a highly controlled environment, at multiple timepoints, after freshwater treatment following initial AGD diagnosis.

## 2. Materials and Methods

### 2.1. Fish Husbandry

Atlantic salmon reared on a land-based freshwater hatchery in the west of Ireland were transferred to a land-based indoor marine recirculating facility at the Marine and Freshwater Research Centre (MFRC) at the Galway-Mayo Institute of Technology (GMIT) in Galway, Ireland following smoltification.

Salmon smolts (*n* = 35), average weight 80 g, were maintained in circular black 1000 L tank with the following conditions: water temperature 12 °C, artificial salinity of 30 parts per thousand (ppt) (Coral Pro salt, Red Sea), light cycle of 12 h light, 12 h dark. Fish were fed a commercial salmon diet at 1% body weight per day. This project was authorized by the Health Products Regulatory Authority (HPRA) in Ireland under project authorization number AE19137/P003, in compliance with the requirements under Directive 2010/63/EU transposed into Irish law by the European Union (Protection of Animals Used for Scientific Purposes) Regulations 2012 (S.I. No 543 of 2012 as amended).

### 2.2. Paramoeba Perurans Culture

*P. perurans* were harvested from naturally infected salmon, sourced from a marine site in the west of Ireland in September 2017, and were cultured on 0.1 malt yeast agar plates in 30 ppt salinity seawater as previously described [20]. Cultures underwent 16 passages before use in inoculation. The identity of the amoebae was confirmed by real-time polymerase chain reaction (PCR), as described in [21], at the Marine Institute, Galway, Ireland.

### 2.3. Paramoeba Perurans Challenge and Sample Collection

**Figure 1:**
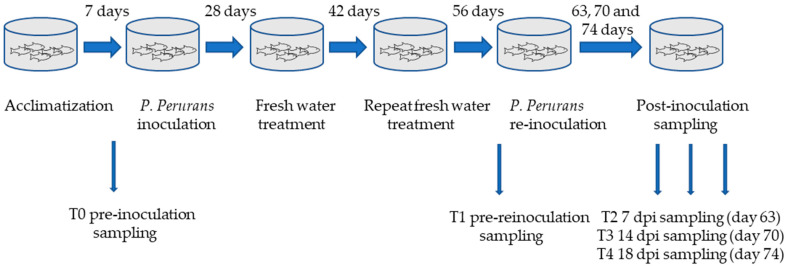
Experimental design of trial investigating the host response to repeat infection with *P. perurans.* Fish were acclimatized for 7 days. Immediately prior to the initial *P. perurans* inoculation, naïve control fish were sampled (T0). AGD was confirmed by gill score analysis 21 days after inoculation and fish were treated by freshwater immersion. Some features of AGD persisted 14 days after freshwater treatment, and the freshwater immersion was repeated. Fish health was confirmed by gross gill analysis 14 days after the second freshwater treatment, and fish were sampled prior to re-infection with *P. perurans* (T1). Fish were sampled at 7 (T2), 14 (T3) and 18 (T4) days post re-infection.

Following an acclimatization period of 7 days, naïve control gill samples (T0) were taken for proteomic analyses. Thirty-five fish were challenged with *P. perurans* (500 amoebae/L) in a volume of 300 L for 4 h with oxygen saturation, and fish behavior and welfare were closely monitored. A further 35 control fish were also held at 300 L for 4 h. The control and treatment fish were moved into new tanks with containing 1000 L seawater. The identity of the amoebae was confirmed by real-time polymerase chain reaction (PCR), as before. Three weeks after inoculation, freshwater treatment was administered by immersion in freshwater tanks for 4 h. Fish were allowed to re-acclimatize and 14 days after freshwater treatment the presence of *P. perurans* was assessed by diagnostic qPCR. The detection of *P. perurans* DNA at this time deemed a second freshwater treatment necessary. A further 14 days after the second freshwater treatment, fish were sampled (T1) and all qPCR samples were negative for the presence of *P. perurans* DNA at this time. The remaining fish were re-inoculated with *P. perurans* (2000 amoebae/L), and fish welfare was monitored throughout as previously described. Further post re-infection samples were taken 7 (T2), 14 (T3), and 18 (T4) days post re-infection (dpi) (Figure 1). For sampling, fish were euthanized by overdose of anesthetic (400 mg L^−1^ tricaine methane sulfonate (MS-222) and when necessary, gill score (GS) was assessed in accordance with the standard commercial protocol adapted from Taylor et al. [4]. This GS system categorizes gill infection across a range of levels from 0, which signifies no visible sign of infection, to 5, which indicates extensive areas of visible lesions across the gill surface. For gill proteomic analysis, the 2nd right gill arch from each fish was excised, the arch cartilage removed, and the remaining gill filaments snap frozen in liquid nitrogen and kept at −80 °C until required.

### 2.4. Proteomics FASP

Gills were weighed, and tissue lysis was performed, on ice, by sonication (4 × 30 s cycles at 25% amplitude; Fisherbrand™ Q500 Sonicator, Waltham, MA, USA), in a 50 mM Tris, 1% SDS lysis buffer. Protein quantification was carried out using the Pierce BCA assay (Thermo Scientific, Waltham, MA, USA) and 300 µg of protein were incubated with 0.1 M DTT at 95 °C for 10 mins. Sample clean up and digestion steps were carried out using the FASP method [22]. Briefly, samples were mixed with UA buffer (8 M urea, 100 mM Tris-HCl pH 8.9) and contaminants and salts were removed by repeated ultrafiltration through 10 kDa Vivacon centrifugal concentrators (Sartorius, Göttingen, Germany).). Samples were incubated for 20 min in darkness with 100 µL 50 mM iodoacetamide in UA buffer, to facilitate blocking of reduced cysteine residues. After further washes with UA and 50 mM ammonium bicarbonate (ABC), trypsin (1 µg per 100 µg protein; Sigma-Aldrich) in 40 µL ABC was added to the samples in the concentrator units. Digestion was performed at 37 °C for 4 h. The resulting peptide fragments were washed with ABC and digestion was halted by the addition of 5 µL 50% acetic acid. The eluates were desalted on C18-Stage pipette tips (Sigma-Aldrich, St. Louis, MO, USA) [23], dried in a vacuum concentrator (Eppendorf Vacufuge concentrator 5301, Sigma-Aldrich) and stored at −20 °C until mass spectrometry analysis.

### 2.5. Mass Spectrometry

The samples were analyzed by the Mass Spectrometry Resource (MSR) at University College Dublin on a Thermo Scientific Q Exactive mass spectrometer connected to a Dionex Ultimate 3000 (RSLCnano, Waltham, MA, USA) chromatography system. Peptides were separated on C18 home-made column (C18RP Reposil-Pur, Ammerbuch, Germany, 100 × 0.075 mm × 3 μm) over 60 min at a flow rate of 250 nL/min with a linear gradient of increasing acetonitrile (ACN) from 1% to 27%. The mass spectrometer was operated in data dependent mode; a high resolution (70,000) MS scan (300–1600 *m*/*z*) was performed to select the twelve most intense ions and fragmented using high energy C-trap dissociation for MS/MS analysis.

### 2.6. Data Processing and Bioinformatics

Raw data from the Q-Exactive were processed using the MaxQuant [24,25] (version 1.6.4.0) incorporating the Andromeda search engine [26]. To identify peptides and proteins, MS/MS spectra were matched against Uniprot Salmo Salar database (2019_04) containing 82,390 entries. All searches were performed using the default setting of MaxQuant, with trypsin as specified enzyme allowing two missed cleavages and a false discovery rate of 1% on the peptide and protein level. The database searches were performed with carbamidomethyl (C) as fixed modification and acetylation (protein N terminus) and oxidation (M) as variable modifications. For the generation of label free quantitative (LFQ) ion intensities for protein profiles, signals of corresponding peptides in different nano-HPLC MS/MS runs were matched by MaxQuant in a maximum time window of 1 min [27]. The mass spectrometry proteomics data have been deposited to the ProteomeXchange Consortium via the PRIDE [28] partner repository with the dataset identifier PXD024096.

### 2.7. Data Analysis

Data were processed using the Perseus (v. 1.6.7.0) data analysis suite (https://maxquant.net/perseus/ (accessed on 22 April 2020)). Label free quantitative (LFQ) ion current intensities were transformed (log_2_). Imputation was carried out to replace missing values with numbers drawn from a normal distribution. A Student’s *t*-test was performed to determined statistical significance (*p* < 0.05). For visualization using heat maps, data were z-score normalized. Fold change values were obtained by calculating the difference between the two compared conditions of the mean log_2_ transformed values, and the Student’s *t*-test *p*-value. Pathway enrichment analysis was performed using the ClueGo (v2.5.4) [29] and Cluepedia (v1.5.7) [30] plugins in Cytoscape (v3.7.1) [31] with the *Salmo salar* (8030) marker set. The gene ontology biological process, consisting of 22,097 genes, was used [32,33]. GO tree levels (min = 3; max = 8) and GO term restriction (min#genes = 3, min% = 4%) were set and terms were grouped using a Kappa Score Threshold of 0.4. The classification was performed by the two-side hypergeometric statistic test, and the probability value was corrected by the Bonferroni method (Adjusted % Term *p*-value < 0.05) [29].

## 3. Results

### 3.1. Diagnostic qPCR and Gill Score Analysis

**Figure 2:**
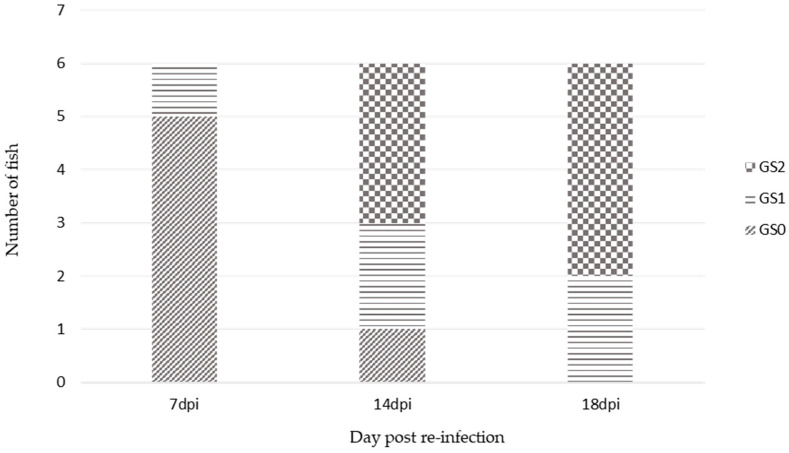
Distribution of gill scores from fish sampled 7 days (T2), 14 days (T3), and 18 days (T4) post re-infection.

Gross assessment of gill health two weeks after the initial freshwater treatment demonstrated persistent mild to moderate gill pathology in 5 of the 6 fish and diagnostic qPCR confirmed the presence of amoeba in gill samples of 6 fish at this timepoint. The freshwater treatment was repeated and after a further 2-week period, qPCR results were negative for *P. perurans*. Seven days post re-infection, only 1 fish of the 6 sampled exhibited GS1. At 14 dpi 2 of the 6 fish sampled had reached GS1 and 3 fish were GS2. By 18 dpi 4 fish were GS2, and the remaining 2 fish were GS1 (Figure 2).

### 3.2. Proteomic Analysis

**Figure 3:**
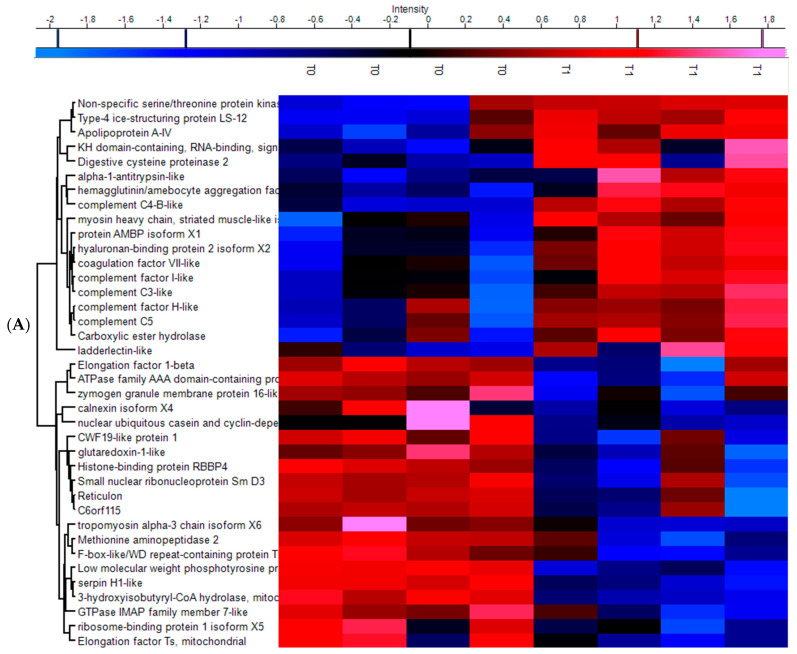

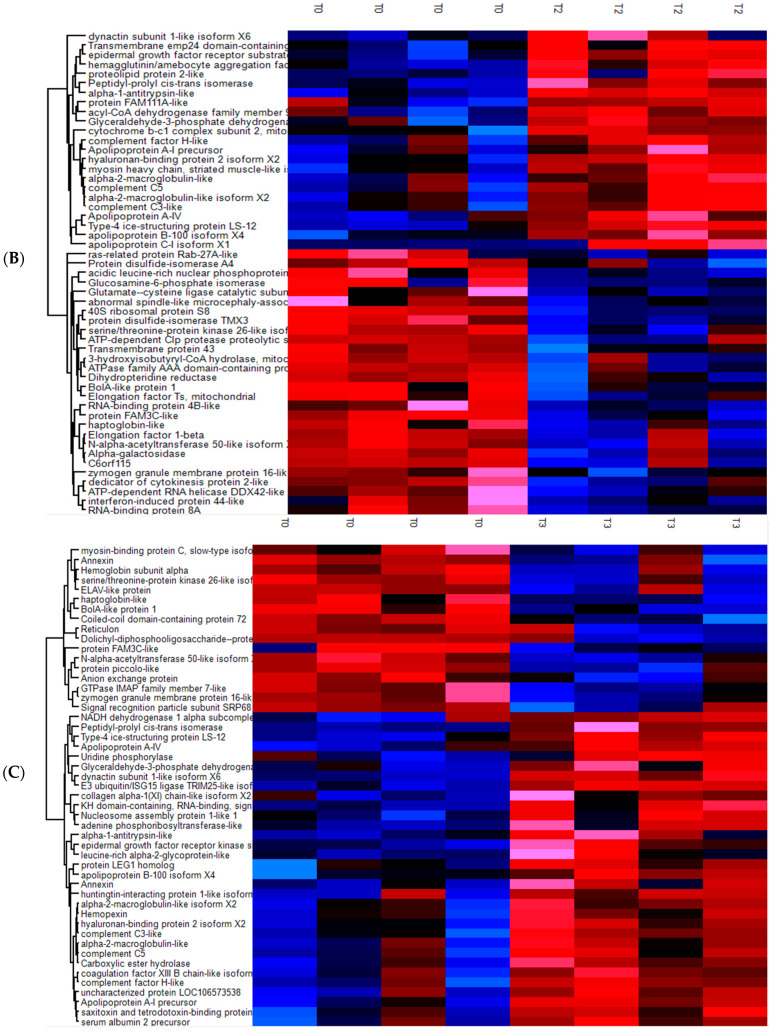

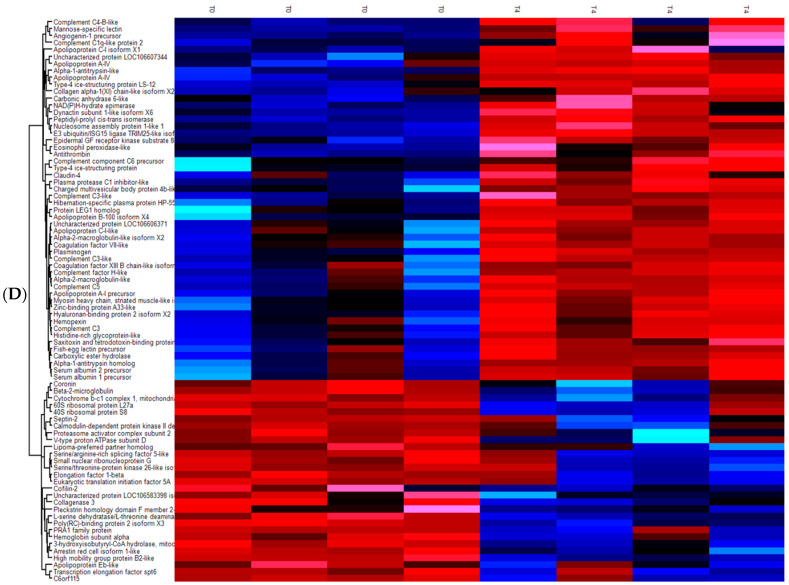
Heatmap plot of the proteins exhibiting *t*-test differences of at least +/− 1.5 in (**A**) control (T0) and post freshwater treatment samples (T1), (**B**) control (T0) and 7 days post re-infection samples (T2), (**C**) control (T0) and 14 days post re-infection samples (T3) and (**D**) control (T0) and 18 days post re-infection samples (T4) after LFQ values were z-score normalized. Hierarchical clustering was performed using Euclidian distance and average linkage using the Perseus software.

In excess of 4000 proteins were initially identified by mass spectrometry. Upon filtering based on matching more than one peptide in each case and performing Student’s *t*-test statistics on normalized and imputed data, 209 proteins were found to be differentially expressed at T1, 225 proteins at T2, 329 proteins at T3, and 288 proteins at T4 when compared to naïve control expression (T0). Between 14% and 28% of these proteins exhibited a *t*-test difference of at least +/− 1.5, across the 4 timepoints (T1, T2, T3, and T4). Heatmap plots of the proteins exhibiting *t*-test differences of at least +/− 1.5 between control (T0) and post freshwater treatment samples (T1) and three post re-infection timepoints (T2, T3, and T4) are displayed in Figure 3A–D. The ClueGO analysis of the key biological processes linked to those same proteins is displayed in Supplemental Figure S1A,B. *t*-test difference values in excess of +/− 1.5 across all timepoints for proteins involved in immunity are displayed in Table 1.

## 4. Discussion

The gravity of AGD in terms of fish mortality alongside the lack of prophylactic measures and the high rate of re-infection means that AGD is one of the most significant health and economic threats in salmon aquaculture. The frequency of infection recurrence, which may necessitate multiple freshwater immersion treatments within one life cycle in the industry setting, will contribute to an elevated stress response in the fish. The resultant increase in plasma cortisol has been shown to suppress immunity, which can contribute to increased fish susceptibility and further exacerbate the issue of re-infection [34,35]. This work attempts to try and elucidate the response of fish re-inoculated *P. perurans* and to determine whether an adaptive response is initiated. While some studies have investigated the gill transcriptomic profile after multiple inoculations [16], and the gill and skin mucus proteomic profiles after repeated *P. perurans* infection [36], here the gill, as the site of amoebic attachment, was chosen as an appropriate tissue for whole proteome analysis. As demonstrated, several proteins with roles in the immune response were found to demonstrate differential expression, when compared to T0 naïve control fish. Some of these proteins were previously described in the proteomic analysis of gill mucus, while others have not been discussed previously in the context of AGD.

The range of complement factors observed to be differentially expressed in the gill upon re-infection reflects the significance of these fundamental components in the host immune response. Upregulation of complement 3, 4, and 5 persisted across all sampling points of re-infection. Complement factor I (CFI) negatively regulates the complement system by cleavage of the C3b and C4b complement factors [37] while plasma protease C1 inhibitor inhibits the alternative complement pathway [38]. Though both were upregulated at some timepoints in this study, they were elevated either prior to re-inoculation, or at a much later stage of the infection. However, complement factor H (CFH), the only complement inhibitory factor to remain elevated throughout this study, is particularly interesting in the context of AGD. CFH binds to complement C3b and blocks the downstream alternative pathway. Crucially, in pathogenic microbe studies in humans, pathogen expression of host-like glycans facilitates pathogen binding to complement factor H, which in turn stimulates host immune suppression as a strategy for microbe survival [39,40,41]. In a previous study (data not published), our group detected a similar upregulation of CFH expression in gill tissue and serum of salmon which had experienced a single infection with *P. perurans*. In that case the upregulation of CFH was apparent in the context of a subdued immune response to infection. It is therefore interesting to note that in both the previous study, which demonstrated a limited immune response to a single *P. perurans* infection, and this study, which demonstrated a more robust immune response to *P. perurans* re-infection, the elevation of CFH remains consistent. This protein and its potential role in facilitating microbial survival in AGD must therefore be given further consideration.

In addition to the role of apolipoproteins in lipoprotein structure and metabolism, their diverse and context dependent roles in innate immunity are becoming increasingly apparent. Apolipoprotein A-I (ApoA-I) and Apolipoprotein C-I (ApoC-I) have been implicated in the binding and neutralization of bacterial lipopolysaccharide (LPS) [42] and the facilitation of LPS induced inflammation in human lung [43] respectively. The upregulation of ApoA-I and two isoforms of ApoC-I upon *P. perurans* re-infection in this study may indicate an immuno-stimulatory response to gill colonization. However apolipoprotein A-IV (ApoA-IV) can negatively impact on leukocyte recruitment to the site of infection or injury [44]. Further, ApoA-IV and ApoA-I have been identified as immune-inhibitory proteins which can impact eosinophil induced inflammation and T-cell signaling respectively [45]. The elevation of these proteins suggest that the expression of the apolipoproteins much be considered carefully in this context. Whether ultimately positively or negatively impacting upon the host immune response, the upregulation of ApoA-I was modest prior to re-infection and showed a more substantial increase in expression with disease progression. The elevation of apoA-IV, however, was detected in fish in the T1 cohort, post freshwater treatment, and prior to re-infection, which implies that the differential expression of this protein must not only be considered in the context of gill colonization. Apolipoprotein B-100 (Apo B-100) predominantly functions as a lipid binding structural protein. It has however been implicated in the limiting of bacterial invasion in *Staphylococcus aureus* infection. Apo B-100 is thought to impact the accessory gene regulator quorum sensing system, which facilitates bacterial adaptation to environmental changes due to population density alterations [46]. *Staphylococcus aureus* colonizes mucosal and epithelial tissue and has the capacity to infiltrate further. Several members of the *Staphylococcaceae* family have been identified on the gills of AGD-affected fish [47]. It is feasible that the significant and persistent upregulation of the apolipoprotein B-100 isoform X4 protein upon re-infection may point to a similar defense mechanism operating in the case of *P. perurans* colonization of gill tissue.

Hyaluronan (HA) is a glycosaminoglycan which is found predominantly in the lung and has been shown to have stimulatory or inhibitory effects on lung pathology, in a manner which is dependent on molecular weight of the hyaluronan and binding of associated proteins. Proinflammatory cytokines, such as TNFα, IL-1β, and LPS induce HA production [48] and HA has been associated with an increase in Muc5 expression in response to oxidative stress in the lung [49]. However, Hyaluronan-binding protein 2 isoform X2, which is persistently upregulated in the current study, hinders HA mediated endothelial cell barrier protection and facilitates LPS induced endothelial barrier disruption in murine acute lung injury models [50].

Alpha-1-antitrypsin (A1AT), a member of the serine protease inhibitor family, is a potent neutrophil elastase inhibitor, which acts to protect lung tissue from excessive proteolytic damage in human models [51]. More recently A1AT has been shown to exert effects on myriad other proteases, and is fundamental for maintaining a fine balance between sufficient protease activity for host immune response and the prevention of uncontrolled tissue injury [52]. Long identified as part of the acute phase immune response, A1AT has since been recognized as having an important role in identifying appropriate responses to legitimate dangers, and avoiding aberrant tissue damage due to over-excitation of inflammatory responses [53]. Protein AMBP isoform X1 is a precursor of the inter-alpha-trypsin inhibitor proteins, α1-microglobulin and bikunin, which, among a diverse range of other functions, exhibit similar serine protease inhibitory functions to A1AT [54]. However, the elevated expression of one of the isoforms of A1AT, and protein AMBP occurs in advance of re-inoculation, and cannot therefore only be considered in the context of *P. perurans* infection. Many isoforms of the AMBP gene products have been localized to lung tissue [55] and one of the major roles of the inter-alpha-trypsin inhibitor proteins is the production of HA complexes. As discussed, HA has been implicated in lung pathology, but also in wound healing in lung tissue [56]. Given that in this study, fish were initially inoculated with *P. perurans*, and subsequently submitted to two cycles of freshwater treatment before post-freshwater sampling, the elevation of the protein AMBP isoform X1 could be interpreted as an indicator of wound healing which would be expected in fish experiencing AGD recovery.

Alpha-2-macroglobulin is also a broad spectrum protease inhibitor which, in addition to its effect on host proteases, confers protection by deactivating pathogen proteases [57]. Both alpha-2-macroglobulin isoforms detected in the gill at various timepoint post-re-infection may be indicative of a host immune response to local colonization.

The proteasome activator complex subunit 2, previously found to be upregulated in transcriptomic analyses of early stage AGD after single *P. perurans* inoculation [58] and other salmon infections [59] has a role in antigen processing by upregulating MHC class 1 presentation. Here protein expression is undetected prior to 18 dpi, at which point proteasome activator complex downregulation is evident. Similarly the downregulation of GTPase IMAP family member 7-like, which has a role in lymphocyte survival [60], is indicative of a suppression of some elements of immunity, however in this case the down regulation was evident before re-infection. Conversely, in this study, the hemagglutinin/amebocyte aggregation factor which has an established role in the mechanism of self/non- self-recognition [61] and E3 ubiquitin/ISG15 ligase TRIM25-like isoform X6, which has a role in innate anti-viral immunity [62] were both found to be upregulated at the early and later timepoints respectively.

Plasminogen is the precursor of plasmin, a broad range serine protease, but has numerous other functions which are highly context dependent. The minor pre-re-infection elevation of plasminogen expression and the more substantial increase at 18 days post re-infection may be indicative of inflammation stimulation, or suppression or indeed complement inhibition, extracellular matrix degradation to facilitate cell migration or leukocyte recruitment to the site of injury. However the concurrent upregulation of histidine-rich glycoprotein-like, coagulation factor VII-like and coagulation factor XIII B chain-like isoform X1, all of which are pro-thrombotic [63], may support the role of plasminogen in coagulation in this case. The diversity of functions attributed to plasminogen suggest it should be considered further in the context of AGD.

Heme release from ruptured red blood cells has been shown to elicit inflammation, through various mechanisms including presentation of LPS to immune cells [64], a process which is suppressed by hemopexin [65]. The context of this suppression is interpreted as protective against tissue damage in response to excessive or prolonged inflammation, or toxicity due to heme accumulation [66]. As an additional defense mechanism, hemopexin scavenging of free heme restricts pathogenic nutrition [67]. In previous *salmo salar* microarray studies hemopexin gene expression was found to be downregulated in *Piscirickettsia salmonis* [68] and *Aeromonas salmonicida* inoculations [69] in earlier stage single infections. The upregulation of two isoforms of hemopexin observed in this study are at the later stages of re-infection and are likely to be in response to prolonged inflammation.

Type-4 ice-structuring protein LS-12 (LS-12), an antifreeze protein, the primary role of which is to lower the freezing point of blood and inhibit the formation of ice crystals, is one of the most profoundly elevated proteins in freshwater treated gills in this study. Interestingly, LS-12 possesses a 20% sequence homology to various members of the apolipoprotein family, which have been implicated in organogenesis and embryonic morphogenesis in Zebrafish [70]. Limited information exists on the expression of LS-12 in the context of immune response or infection. However, a tick-derived antifreeze protein was shown to exert anti-virulence properties in mouse models challenged with bacteria [71]. In this study the initial elevation in expression detected in the freshwater treated gills at T1 is further stimulated by re-infection, with levels continuing to increase with disease progression. Whether the activity of the LS-12 protein mirrors the functions of those apolipoproteins which share sequence homology, or whether its function may be linked to an adaptive host immune response remains to be determined. To date this protein has not been reported in the context of AGD and warrants further investigation as a potential protein of interest in the adaptive host response.

Lectins have an important role in innate immunity in teleosts. Members of the lectin family act as pathogen recognition molecules, which bind to the surfaces of pathogens, thus facilitating their recognition and clearance [72]. Fish-egg lectin (FEL), a member of the rhamnose-binding lectin family is traditionally associated with immune defense in early stages of fish development, and is predominantly found in oocytes, although some family members have been isolated from fish skin mucus and various organs and cells of the immune system [73]. While in some fish species, FEL are shown to bind to a wide variety of both gram positive and gram negative bacteria [74], pertinently for this study, FEL are thought to exhibit highly specific binding to rhamnose [73]. Rhamnose is a monosaccharide and is one of the major constituents of the carbohydrate portion of the high molecular weight antigen in virulent *P. perurans* [75]. The elevation of the fish egg lectin precursor protein at the latest timepoint in this study is suggestive of a response to the presence of infectious *P. perurans*, and this is the first time FEL has been implicated in the host response in AGD.

Taken together, the data here demonstrate a host immune response to repeat inoculation with *P. perurans*. However, many of the proteins identified as exhibiting differential expression in response to re-inoculation are part of the innate immune response, and there are few indicators of an adaptive immune response in the gill in this study. Nonetheless, many of the proteins identified are novel in the context of AGD and their expression profiles suggest that their roles in the response to disease development and progression in single or multiple infections warrant further investigation.

## Figures and Tables

**Table 1:** Immune-related proteins differentially expressed in the gills of *P. perurans* inoculated salmon after freshwater treatment for initial *P. perurans* infection (T1) and at three timepoints post re-infection (T2, T3, and T4). Proteins were identified by mass spectrometry and analysis was performed by Max Quant and Perseus computational platforms. For each time point protein expressions were compared to naïve controls (T0). Reported fold change values were obtained by calculating the difference between the mean log_2_ transformed values for the two compared conditions (*p* value < 0.05).

Name	Uniprot Id	T0 v T1	T0 v T2	T0 v T3	T0 v T4	No. of Peptides
Alpha-1-antitrypsin homolog	A0A1S3SAE4			1.34	1.56	18
Alpha-1-antitrypsin-like	A0A1S3PJ46	1.90	2.35	2.40	2.56	5
Alpha-2-macroglobulin-like	A0A1S3SY27		1.67	1.59	2.08	37
Alpha-2-macroglobulin-like isoform X2	A0A1S3SY28		2.09	1.94	2.22	19
Apolipoprotein A-I precursor	B5XBH3	1.32	1.66	2.28	2.28	29
Apolipoprotein A-IV	B5X8U6	2.57	3.47	4.09	4.58	15
Apolipoprotein B-100 isoform X4	A0A1S3M896		3.11	2.62	3.60	61
Apolipoprotein C-I isoform X1	A0A1S3RYY8		3.23		2.63	4
Apolipoprotein C-I-like	A0A1S3N6L4				1.53	7
Coagulation factor VII-like	A0A1S3MXK4	1.80			1.53	15
Coagulation factor XIII B chain-like isoform X1	A0A1S3QZV0	1.41		1.59	1.53	15
Complement C3	A0A1S3QRL5			1.30	1.58	96
Complement C3-like	A0A1S3L7F1	1.80	1.66	1.99	2.35	35
Complement C3-like	A0A1S3PMF1		1.40		2.01	23
Complement C4-B-like	A0A1S3NRS7	2.85			1.77	9
Complement C5	A0A1S3PRG9	1.96	1.73	1.93	2.43	41
Complement factor H-like	A0A1S3QR20	1.51	1.89	1.81	2.02	47
Complement factor I-like	A0A1S3QGL8	2.11				8
E3 ubiquitin/ISG15 ligase TRIM25-like isoform X6	A0A1S3L7B4			2.80	2.52	3
Fish-egg lectin precursor	B5X7U2				1.96	6
Gtpase IMAP family member 7-like	B9ENC9	−2.33		−1.93		4
Hemagglutinin/amebocyte aggregation factor-like isoform X1	A0A1S3MZH7	2.38	1.71			4
Hemopexin	A0A1S3MVQ2			1.53	1.43	22
Hemopexin	A0A1S3PQV6				1.73	28
Histidine-rich glycoprotein-like	A0A1S3KK24				1.54	20
Hyaluronan-binding protein 2 isoform X2	A0A1S3N344	2.12	1.69	1.83	2.18	13
Plasma protease C1 inhibitor-like	A0A1S3SJI8	1.37			1.55	13
Plasminogen	A0A1S3MAB2	1.32			1.84	19
Proteasome activator complex subunit 2	B5X6E1				−1.58	10
Protein AMBP isoform X1	A0A1S3LTS1	1.50				10
Type-4 ice-structuring protein	B5X6Y1				2.08	4
Type-4 ice-structuring protein LS-12	B5XDA4	3.19	4.76	4.79	5.88	9

## Data Availability

Data are available via ProteomeXchange with identifier PXD024096.

## Supplementary Materials

The following are available online at https://www.mdpi.com/article/10.3390/microorganisms9050993/s1, Figure S1: Network (A) and Bar- graph (B) representations of ClueGO analysis of the key biological processes linked to those proteins which demonstrated a *t*-test difference of at least +/− 1.5 between T0 and T4.
