# Supplementary material for: Host Response of Atlantic Salmon (Salmo salar) Re-Inoculated with Paramoeba perurans
Source: Microorganisms. 2021 May 5;9(5):993. doi: 10.3390/microorganisms9050993 (PMC8147987; doi:10.3390/microorganisms9050993)
Supplement: Supplementary file 1 [file microorganisms-09-00993-s001.zip › microorganisms-1176061-supplementary.pdf]

A

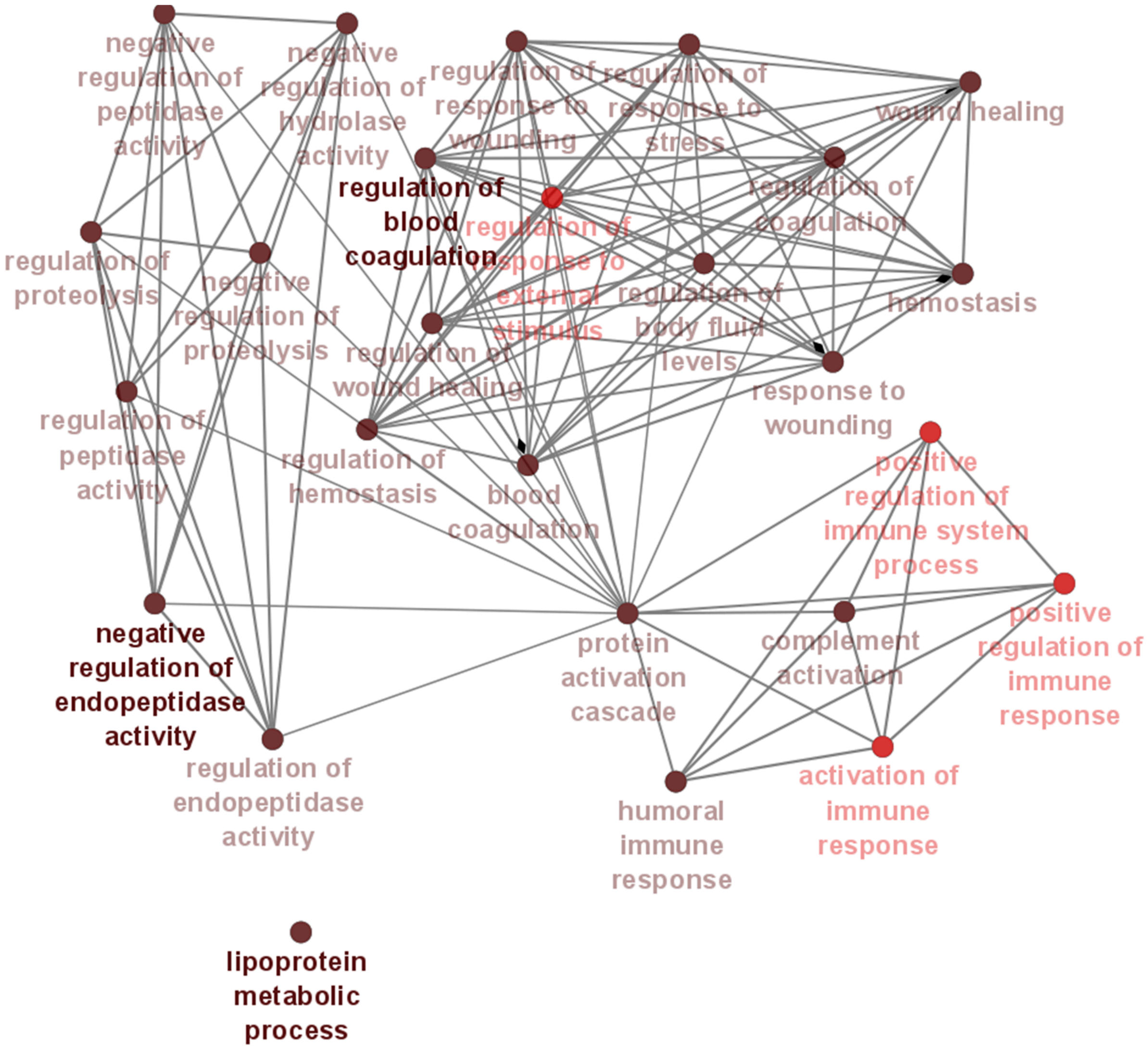

negative regulation of peptidase activity
negative regulation of hydrolase activity
regulation of response to wounding
regulation of response to stress
wound healing
regulation of blood coagulation
regulation of response to external stimulus
regulation of coagulation
regulation of proteolysis
negative regulation of proteolysis
regulation of body fluid levels
hemostasis
regulation of wound healing
response to wounding
regulation of peptidase activity
regulation of hemostasis
blood coagulation
positive regulation of immune system process
positive regulation of immune response
negative regulation of endopeptidase activity
protein activation cascade
complement activation
regulation of endopeptidase activity
activation of immune response
humoral immune response
lipoprotein metabolic process

B

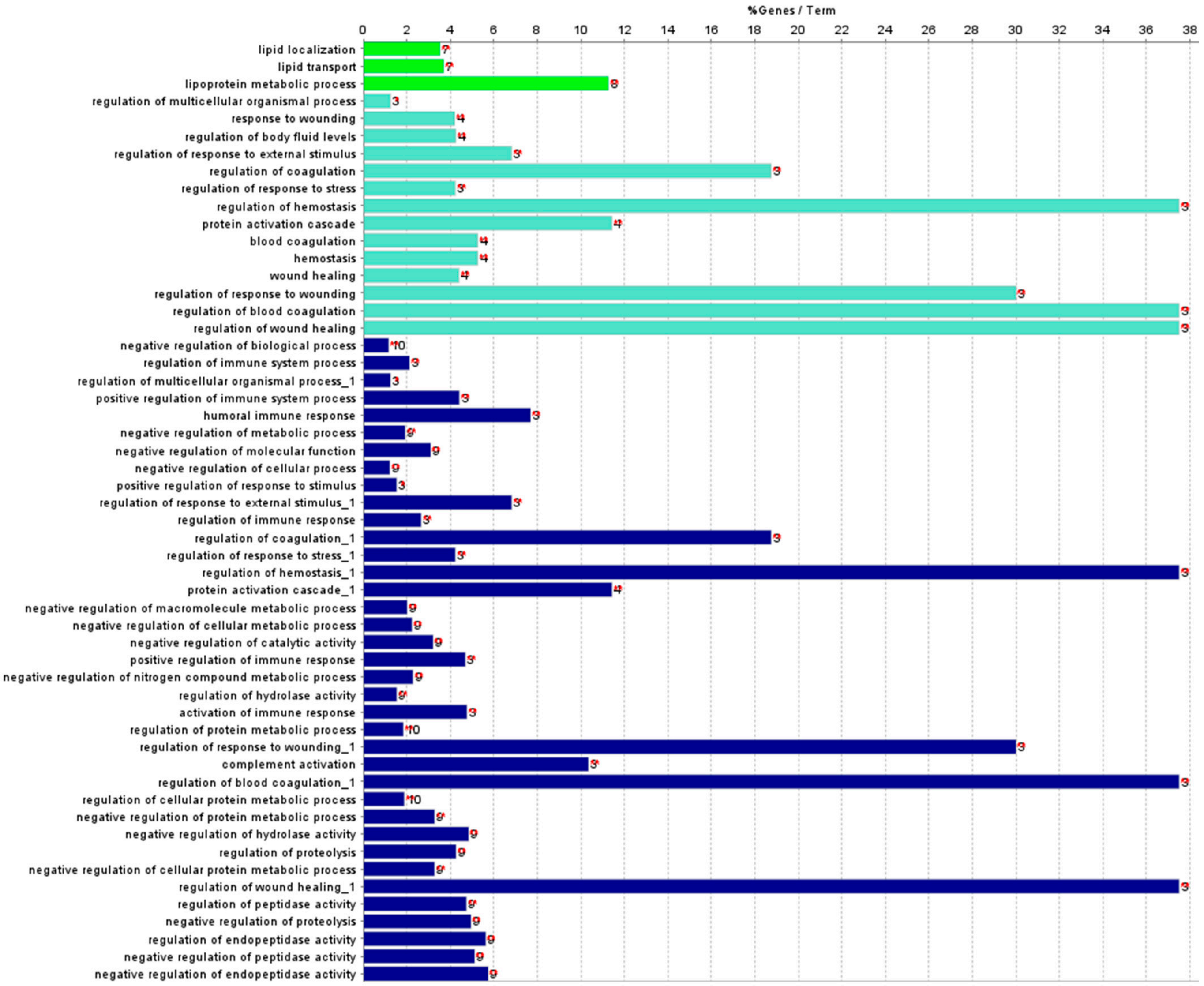

Figure S1. Network (**A**) and Bar- graph (**B**) representations of ClueGO analysis of the key biological processes linked to those proteins which demonstrated a t-test difference of at least +/- 1.5 between T0 and T4.
